# Self-Sensing Control for Soft-Material Actuators Based on Dielectric Elastomers

**DOI:** 10.3389/frobt.2019.00133

**Published:** 2019-12-13

**Authors:** Thorben Hoffstadt, Jürgen Maas

**Affiliations:** Mechatronic System Laboratory, Institute of Machine Design and Systems Technology, Technische Universität Berlin, Berlin, Germany

**Keywords:** dielectric elastomers, self-sensing, control, soft material actuator, extended Kalman filter, stack-actuator, flyback-converter

## Abstract

Due to their energy density and softness that are comparable to human muscles dielectric elastomer (DE) transducers are well-suited for soft-robotic applications. This kind of transducer combines actuator and sensor functionality within one transducer so that no external senors to measure the deformation or to detect collisions are required. Within this contribution we present a novel self-sensing control for a DE stack-transducer that allows to control several different quantities of the DE transducer with the same controller. This flexibility is advantageous e.g., for the development of human machine interfaces with soft-bodied robots. After introducing the DE stack-transducer that is driven by a bidirectional flyback converter, the development of the self-sensing state and disturbance estimator based on an extended Kalman-filter is explained. Compared to known estimators designed for DE transducers supplied by bulky high-voltage amplifiers this one does not require any superimposed excitation to enable the sensor capability so that it also can be used with economic and competitive power electronics like the flyback converter. Due to the behavior of this converter a sliding mode energy controller is designed afterwards. By introducing different feed-forward controls the voltage, force or deformation can be controlled. The validation proofs that both the developed self-sensing estimator as well as the self-sensing control yield comparable results as previously published sensor-based approaches.

## 1. Introduction

Entirely soft-bodied robots exploit the full potential of robotic systems in terms of safe human-machine-interactions and, thus, are in the scope of research. However, novel mechanical designs in conjunction with smart and soft materials as well as innovative approaches for modeling and the development of control strategies to handle such a highly sophisticated robot species are necessary (Navarro et al., 2013; Robla-Gomez et al., 2017). Due to their behavior that resembles human muscles, dielectric elastomers (DEs) are a promising approach that could pave the way for soft-bodied robots. As a DE transducer consists of a very thin, elastomeric dielectric film covered with compliant electrodes, its behavior can be described by a shape varying capacitor.

By applying a voltage *v*_*p*_ to the electrodes of the DE transducer with permittivity ε_0_ · ε_r_ and thickness *d* the resulting electrostatic pressure

(1) $$\sigma_{el}=\varepsilon_{0}\;\cdot \;\varepsilon_{\text{r}}\;\cdot \;{\left(\frac{v_{\text{p}}}{d}\right)}^{2}$$

compresses the elastomer. This pressure is used to operate a DE transducer in actuator mode. However, if the change of the transducer's capacitance is detected that is caused by its deformation, a simultaneous operation as sensor is enabled. If the deformation dependency of the capacitance is known, the mechanical transducer state can be determined. By exploiting this self-sensing capability, soft and smart transducers can be realized that do not require additional external sensors and, thus, can be comparably easy integrated into various applications not limited to soft robotics.

For various types of DE transducers different approaches to control their displacement or force (Maas et al., 2011; Sarban and Jones, 2012; Rizzello et al., 2015; Wilson et al., 2016; Hoffstadt and Maas, 2017, 2018b) or to use them for active vibration attenuation (Dubois et al., 2008; Kaal and Herold, 2011; Sarban, 2011) have been presented previously. Within these approaches the control variables are directly measured with external sensors, so that the DE transducer is only operated as actuator. Due to the additional sensor, these controls are referred to as sensor-based control schemes.

**Figure 1:**
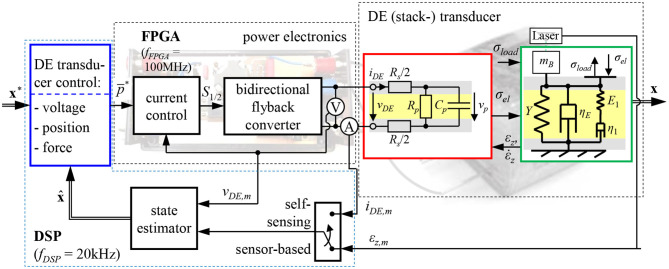
Fundamental structure of the proposed closed-loop position control for DE stack-actuators fed by a bidirectional flyback-converter.

Within this paper, the focus is given on the development of a model-based self-sensing control for DE transducers that allows to control the voltage, force and deformation of the transducer without measuring any mechanical quantities. Figure 1 gives an overview of the overall developed control circuit.

As shown in the center and on the right hand side of Figure 1 the terminal voltage *v*_DE_ and current *i*_DE_ have to be measured to enable the combined actuator-sensor-operation. In order to determine the mechanical state based on these measurement quantities adequate self-sensing algorithms are required. Anderson et al. (2012) summarizes different approaches for this purpose. The goal of most self-sensing algorithms is to identify the capacitance of the DE transducer in a first step and afterwards estimate the deformation and force based on a model or experimentally obtained information about the deformation dependency of the capacitance. For almost all approaches the driving voltage *v*_DE_ is superimposed with a harmonic excitation that is used for the sensor functionality.

Chuc et al. (2008) and Jung et al. (2008) published first frequency domain based approaches by experimentally identifying changes of the electrical impedance of a DE transducer under deformation when it is excited by a harmonic voltage *v*_DE_. Beside the capacitance *C*_p_ they also considered losses in the polymer and the electrode by adding the resistances *R*_s_ and *R*_p_, respectively, see Figure 1.

In Hoffstadt et al. (2014) another model-based identification algorithm in the frequency domain is presented that estimates the electrical parameters of a DE transducer by evaluating the amplitudes of and the phase shift between the superimposed terminal voltage and current. Furthermore, it was shown that the behavior of a DE transducer can be sufficiently modeled by neglecting the parallel resistance *R*_p_ representing losses in the dielectric, if the DE transducer is excited with a comparable high frequency.

The extended Kalman-filter introduced in Hoffstadt and Maas (2018a) estimates the strain of a DE transducer without any superimposed excitation so that it can be used independent of the utilized power electronics. Other approaches in the time domain estimate the charge *q*_p_ of the capacitance *C*_p_. Under further consideration of the measured voltage *v*_DE_ the capacitance *C*_p_ ≈ *q*_p_/*v*_DE_ can be determined (Matysek et al., 2011; Gisby et al., 2013).

Rizzello et al. (2017) developed a self-sensing algorithm based on the recursive least squares (RLS) method. For this purpose, he takes into account the equivalent circuit diagram with three parameters (see Figure 1). In a first step, the parameters of a discrete transfer function describing the behavior of the considered circuit are estimated. As these parameters depend on the electrical parameters, they can be calculated afterwards. For the identification a harmonic excitation signal is superimposed.

Although several self-sensing approaches have been developed only a few closed-loop self-sensing controller designs have been published, so far. Gisby et al. (2011) controls the deformation of a single-layer circular DE transducer by using the already mentioned self-sensing approach (Gisby et al., 2013). Here, the terminal voltage is PWM generated. While the deformation of the DE transducer mainly depends on the mean of this voltage, the included higher harmonics are used to enable the sensor functionality. The manually adjusted proportional gain controller yields comparable low dynamics and accuracy. Therefore, Rosset et al. (2013) extends this controller to a PI-controller, using the same self-sensing approach (Gisby et al., 2013). Here, the parameters of the controller are optimized for one particular operating point of the nonlinear control plant. The derived controller is used to control an optical grid.

Rizzello et al. (2016) systematically combines his RLS-based self-sensing approach (Rizzello et al., 2017) with his robust position controller (Rizzello et al., 2015) to control the deformation of a DE membrane actuator. For the combined actuator-sensor-operation the required driving voltage determined by the controller is superimposed with a harmonic excitation with a high frequency of 1 kHz and an amplitude of 75 V. Compared to the sensor-based control (Rizzello et al., 2015) almost no drawbacks in terms of the accuracy are observed, while the bandwidth of the closed-loop self-sensing control is reduced due to the dynamics of the parameter identification.

Within the referenced publications costly and bulky high-voltage amplifiers were used to feed the DE transducer. However, due to the capacitive behavior of DE transducers voltage-fed current sources are well suited instead of high-voltage amplifiers (Eitzen et al., 2011a). Here, compact and efficient driving electronics can be realized when using switched-mode operated topologies like the bidirectional flyback converter. This converter allows not only to supply the DE transducer with a certain voltage but also to recover the energy stored in the DE transducer when discharging it.

Under consideration of the properties of the bidirectional flyback converter and the DE transducer, we previously published sensor-based position and force controls in Hoffstadt and Maas (2017, 2018b) that use the directly measured deformation as feedback-signal, cf. Figure 1. Within this publication we extend them to a self-sensing controller that is able to universally control the voltage, force or deformation of the DE transducer by just measuring the terminal voltage *v*_DE_ and current *i*_DE_. For this purpose, in the following section 2 the considered control plant comprising a DE stack-transducer (Maas et al., 2015) fed by a bidirectional flyback converter (Eitzen et al., 2011b; Hoffstadt and Maas, 2016) is introduced and modeled. The design of the novel self-sensing state and disturbance estimator is presented in section 3. Due to the non-linear behavior of the control plant an extended Kalman-filter (EKF) is used for this purpose (Welch and Bishop, 2001). The developed estimator does not require any superimposed excitation. The subsequently presented controller design (Hoffstadt and Maas, 2017, 2018b) is based on the sliding mode control approach (DeCarlo et al., 1988) as this is well suited for the considered control plant and its characteristic behavior. The self-sensing estimator and control are experimentally validated in section 5. Finally, section 6 summarizes the developed approaches and the result.

## 2. Model of the DE Transducer System

**Figure 2:**
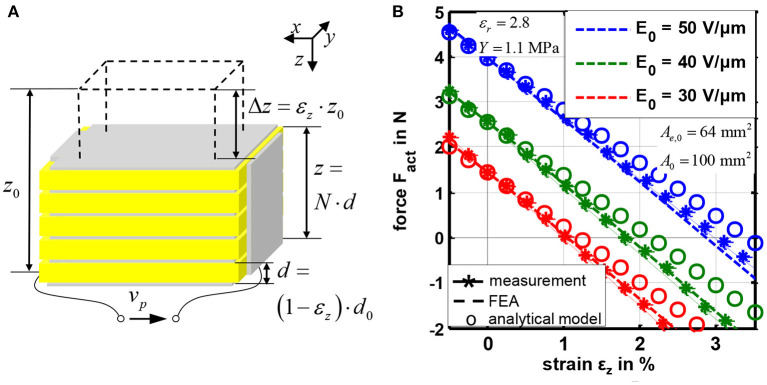
Schematic design **(A)** and static strain-force characteristics **(B)** of the considered silicone-based DE stack-actuators.

Figure 2A shows a schematic representation of the considered DE stack-transducer with *N* layers. This multilayer design is used to scale the deformation Δ*z* in *z*-direction, as one single layer has an initial thickness of only *d*_0_ = 50 μm. Details about the design and the manufacturing were published by Maas et al. (2015). The static strain-force-behavior is shown in Figure 2B. The transducer generates higher tensile forces *F*_act_ at smaller strains ε_*z*_ = Δ*z*/*z*_0_, with *z*_0_ = *N* · *d*_0_. By increasing the initial electric field strength *E*_0_ = *v*_DE_/*d*_0_ the electrostatic pressure according to Equation (1) increases so that higher forces and strains are obtained. The blocking-force *F*_act_(ε_*z*_ = 0) and the no-load strain ε_*z*_(*F*_act_ = 0) represent two characteristic points of the strain-force behavior.

An analytical model for this transducer is published in Hoffstadt and Maas (2015). In Figure 2 the modeled results of the static strain-force behavior are compared with measurement results and a finite element analysis (FEA) published by Kuhring et al. (2015). The analytical model is based on the structure shown on the right of Figure 1. The actuator tension σ_act_ is given by the force equilibrium:

(2) $$\sigma_{\text{act}}=\beta \;\cdot \;\sigma_{\text{el}}-\sigma_{\text{elast}}-\eta_{E}\;\cdot \;{\dot{\varepsilon }}_{z}-E_{1}\;\cdot \;\varepsilon_{E_{1}},\text{ with }\beta =\frac{A_{\text{e}}}{A}.$$

Here, σ_elast_ is the elastic material tension that is calculated using the Neo-Hookean approach with the Young's modulus *Y* to consider the hyperelastic, non-linear material behavior:

(3) $$\sigma_{elast}=\frac{Y}{3}\;\cdot \;\left(\frac{1}{1-\varepsilon_{z}}-{\left(1-\varepsilon_{z}\right)}^{2}\right).$$

Beside this reversible elastic behavior, viscoelastic properties are taken into account with the viscosity η_*E*_ and the Maxwell element with stiffness *E*_1_ and viscosity η_1_. Furthermore, with the area ratio β it is considered that the electrostatic pressure σ_el_ acts only on the area *A*_e_ covered with electrode, while all other tensions are assumed to homogeneously act on the whole transducer area *A* in *z*-direction. Instead of applying Equation (1) for the electrostatic pressure σ_el_, here it is determined depending on the energy *U*_c,diel_ in the electric field of the capacitance *C*_*p*_:

(4) $$\beta \;\cdot \;\sigma_{\text{el}}=\frac{2}{V}\;\cdot \;U_{\text{c},\text{diel}}.$$

The bidirectional flyback converter control proposed in Hoffstadt and Maas (2016) enables three discrete input states in terms of the feeding power $\bar{p}$. Beside an off-state, the DE can be charged and discharged with almost constant power depending on the characteristic energy increment Δ*U*_max_ transfered during every switching period *T*_S_ of the converter:

(5) $$\begin{array}{l} \bar{p}=\{\begin{array}{l} +{\bar{p}}_{\max}=+\frac{\Delta U_{\max}}{T_{\text{S}}},\text{ charging}\\ \;0,\text{ off-state}\\ -{\bar{p}}_{\max}=-\frac{\Delta U_{\max}}{T_{\text{S}}},\text{ discharging} \end{array}\\ \text{with }\Delta U_{\max}=\frac{1}{2}\;\cdot \;L_{\text{m}}\;\cdot \;I_{\text{m},\max}. \end{array}$$

The energy increment Δ*U*_max_ depends on the magnetizing inductance *L*_m_ of the converter and the magnetizing current *I*_m,max_ adjusted by its inner control. Under further consideration of losses *p*_*Re*_ dissipated in the electrode material the power ${\bar{p}}^{\prime }=\bar{p}-p_{Re}$ feeds the capacitance of the DE transducer. With this, the electromechanically coupled behavior of a DE transducer can be modeled based on a power balance yielding the state space representation

(6) $$\begin{array}{l} \dot{x}=\left[\begin{array}{c} {\dot{\varepsilon }}_{z}\\ \frac{V}{m_{\text{B}}\cdot z_{0}^{2}}\;\cdot \;\frac{\sigma_{\text{act}}-\sigma_{\text{load}}}{1-\varepsilon_{z}}\\ {\dot{\varepsilon }}_{z}-\frac{E_{1}}{\eta_{1}}\;\cdot \;\varepsilon_{E_{1}}\\ -2\;\cdot \;U_{\text{c},\text{diel}}\;\cdot \;\left(\frac{{\dot{\varepsilon }}_{z}}{1-\varepsilon_{z}}+\frac{1}{\tau_{\text{p}}}\right) \end{array}\right]+\left[\begin{array}{c} 0\\ 0\\ 0\\ 1 \end{array}\right]\;\cdot \;{\bar{p}}^{\prime },\\ \text{with }x=\left[\begin{array}{c} \varepsilon_{z}\\ {\dot{\varepsilon }}_{z}\\ \varepsilon_{E_{1}}\\ U_{\text{c},\text{diel}} \end{array}\right]. \end{array}$$

Beside the strain ε_*z*_ and the energy *U*_c,diel_ the state vector *x* includes the velocity ${\dot{\varepsilon }}_{z}$ as well as the strain ε_*E*_1__ of the stiffness *E*_1_ of the Maxwell element. Depending on the supplied input power ${\bar{p}}^{\prime }$ and an external load σ_load_ the inner states of the DE transducer with volume *V* and accelerated mass *m*_B_ can be calculated with Equation (6).

For the subsequently developed self-sensing state estimator models describing the strain dependency of the electrical transducer parameters are required, too. The series resistance *R*_*s*_ mainly comprises losses in the contacting of the DE transducer and electrodes that are applied on the initial area *A*_e, 0_ of every layer. It was shown Hoffstadt et al. (2016) that this resistance is almost constant in the relevant range of deformation. In contrast, the capacitance *C*_*p*_ for the *N* layers connected in parallel is given by:

(7) $$C_{\text{p}}=N\;\cdot \;\varepsilon_{0}\;\cdot \;\varepsilon_{\text{r}}\;\cdot \;\frac{A_{\text{e},\text{0}}}{d_{0}}\;\cdot \;\frac{1}{{\left(1-\varepsilon_{z}\right)}^{\kappa }}=C_{\text{p},\text{0}}\;\cdot \;\frac{1}{{\left(1-\varepsilon_{z}\right)}^{\kappa }}$$

The change of the initial capacitance *C*_p, 0_ also depends on the factor κ. In case of an absolutely homogeneous deformation without constraints, κ = 2 would apply. However, due to a passive area around *A*_e_ that is required for insulation purposes, as well as due to stiff mechanical interfaces applied on the top and/or bottom of the transducer, here the factor is slightly decreased to κ = 1.85.

In analogy, the strain dependency of the parallel resistance *R*_p_ reads as follows:

(8) $$R_{\text{p}}=\frac{1}{N}\;\cdot \;\rho_{\text{p}}\;\cdot \;\frac{d_{0}}{A_{\text{e},\text{0}}}\;\cdot \;{\left(1-\varepsilon_{z}\right)}^{\kappa }=R_{\text{p},\text{0}}\;\cdot \;{\left(1-\varepsilon_{z}\right)}^{\kappa }.$$

This resistance represents losses in the dielectric with the specific resistance ϱ_p_.

Although *C*_p_ and *R*_p_ vary with the strain ε_*z*_ the resulting time constant τ_p_ is independent of the strain:

(9) $$\tau_{\text{p}}=R_{\text{p}}\;\cdot \;C_{\text{p}}=\varepsilon_{0}\;\cdot \;\varepsilon_{\text{r}}\;\cdot \;\rho_{\text{p}}=\text{const.}$$

## 3. EKF-Based Self-Sensing Algorithm

In Hoffstadt and Maas (2018a) we already published a self-sensing estimator based on a discrete, extended Kalman-filter that estimates the strain of the DE transducer without superimposed excitation. However, for the closed-loop operation beside the inner states of the transducer also the disturbance σ_load_ has to be estimated. For example, this load tension might result from a collision of an external device or human with a soft-bodied robot equipped with DE transducers. Therefore, a new and extended approach based on Equation (6) is applied here. As mentioned above, the goal is to determine the electromechanical state of the DE transducer based on the measured terminal voltage *v*_DE_ and the current *i*_*DE*_. However, in Equation (6) the energy *U*_c,diel_ represents the electrical state. Therefore, a modification of the model is required to design the self-sensing estimator.

For this purpose, the change of the charge *q*_p_ on the capacitance *C*_p_ is taken into account. It can be calculated under consideration of the current *i*_DE_ and the leakage current *v*_p_/*R*_p_ = *q*_p_/τ_p_, see Figure 1:

(10) $${\dot{q}}_{\text{p}}=i_{\text{DE}}-\frac{v_{\text{p}}}{R_{\text{p}}}=i_{\text{DE}}-\frac{1}{\tau_{\text{p}}}\;\cdot \;q_{\text{p}},\text{ with }q_{\text{p}}=C_{\text{p}}\;\cdot \;v_{\text{p}}.$$

As the charge depends on the measured current and the invariant time constant τ_p_ from Equation (9), it is used as input variable *u*_*qv*_ in the following. Furthermore, if instead of the energy *U*_c,diel_ the charge *q*_p_ is considered, the electrostatic pressure can be expressed by:

(11) $$\beta \;\cdot \;\sigma_{\text{el}}=\frac{2}{V}\;\cdot \;U_{\text{c},\text{diel}}=\frac{q_{\text{p}}^{2}}{V\;\cdot \;C_{\text{p}}\left(\varepsilon_{z}\right)},\text{ with }U_{\text{c},\text{diel}}=\frac{1}{2}\;\cdot \;\frac{q_{\text{p}}^{2}}{C_{\text{p}}\left(\varepsilon_{z}\right)}.$$

Additionally, the voltage *v*_p_ across *C*_p_ depends on the terminal voltage *v*_DE_ reduced by the voltage drop *R*_s_ · *i*_DE_ across the series resistance *R*_s_ that is assumed to be constant here:

(12) $$v_{\text{p}}=\frac{q_{\text{p}}}{C_{\text{p}}\left(\varepsilon_{z}\right)}=v_{\text{DE}}-R_{\text{s}}\;\cdot \;i_{\text{DE}}.$$

This voltage will be used as output variable *y*_*qv*_ afterwards.

Beside the mechanical states included in *x* and Equation (6) the external load σ_load_ has to be estimated as disturbance, too. As it represents an unknown disturbance it is assumed (according to Isermann and Munchhof, 2011) that it is constant during one sample time *T* of the discrete EKF implemented on a DSP. By applying ${\dot{\sigma }}_{\text{load}}=0$ in combination with Equations (10)–(12) a fourth order system can be established for the estimation:

(13) $$\begin{array}{l} x_{qv}^{\cdot }=f_{qv}\left(x_{qv},\;u_{qv}\right)=\left[\begin{array}{c} {\dot{\varepsilon }}_{z}\\ \gamma_{1}\;\cdot \;\frac{\sigma_{\text{act}}\left(q_{p}\right)-\sigma_{\text{load}}}{1-\varepsilon_{z}}\\ {\dot{\varepsilon }}_{z}-\frac{E_{1}}{\eta_{1}}\;\cdot \;\varepsilon_{E_{1}}\\ 0 \end{array}\right],\\ \text{with }x_{qv}=\left[\begin{array}{c} \varepsilon_{z}\\ \varepsilon_{z}\\ \varepsilon_{E_{1}}\\ \sigma_{\text{load}} \end{array}\right],\;\gamma_{1}=\frac{V}{m_{\text{B}}\;\cdot \;z_{0}^{2}}\text{ and}\\ y_{qv}=g_{qv}\left(x_{qv},\;u_{qv}\right)=\frac{{\left(1-\varepsilon_{z}\right)}^{\kappa }}{C_{\text{p},\text{0}}}\;\cdot \;q_{\text{p}}=v_{\text{p}}=v_{\text{DE},\text{m}}-R_{\text{s}}\;\cdot \;i_{\text{DE},\text{m}}. \end{array}$$

According to Adamy (2014) the observability of the nonlinear system (13) is given if the determinant of the observability matrix *Q*_B,*qv*_ is not zero. This matrix can be calculated under consideration of the Lie derivatives $L_{f_{qv}}^{i}g_{qv}$, with *i* = 0, …, 3:

(14) $$Q_{\text{B},qv}\left(x_{qv},u_{qv}\right)=\left[\begin{array}{c} \frac{\partial L_{f_{qv}}^{0}g_{qv}\left(x_{qv},u_{qv}\right)}{\partial x_{qv}}\\ \frac{\partial L_{f_{qv}}^{1}g_{qv}\left(x_{qv},u_{qv}\right)}{\partial x_{qv}}\\ \frac{\partial L_{f_{qv}}^{2}g_{qv}\left(x_{qv},u_{qv}\right)}{\partial x_{qv}}\\ \frac{\partial L_{f_{qv}}^{3}g_{qv}\left(x_{qv},u_{qv}\right)}{\partial x_{qv}} \end{array}\right]=\left[\begin{array}{c} \frac{\partial g_{qv}\left(x_{qv},u_{qv}\right)}{\partial x_{qv}}\\ \frac{\partial L_{f_{qv}}^{1}g_{qv}\left(x_{qv},u_{qv}\right)}{\partial x_{qv}}\\ \frac{\partial L_{f_{qv}}^{2}g_{qv}\left(x_{qv},u_{qv}\right)}{\partial x_{qv}}\\ \frac{\partial L_{f_{qv}}^{3}g_{qv}\left(x_{qv},u_{qv}\right)}{\partial x_{qv}} \end{array}\right]$$

Beside material parameters that are different from zero, the determinant of *Q*_B,*qv*_ depends on the charge *q*_p_ and the strain ε_*z*_:

(15) $$\begin{array}{l} \det\;\left(Q_{\text{B},qv}\left(x_{qv},\;u_{qv}\right)\right)=q_{\text{p}}^{4}\;\cdot \;\frac{\gamma_{1}^{2}\cdot \kappa^{4}\cdot E_{1}^{2}}{\eta_{1}\cdot C_{\text{p}}^{4}\left(\varepsilon_{z}\right)\cdot {\left(1-\varepsilon_{z}\right)}^{6}}\neq 0,\\ \text{ for }\varepsilon_{z}<1\text{ and }q_{\text{p}}\neq 0. \end{array}$$

The strain ε_*z*_ is always smaller than one, and thus does not influence the observability. However, the uncharged state with *q*_p_ = 0 is not observable. In contrast for example to piezoelectric materials, this is due to the fact that the DE materials do not contain inherent dipoles causing a charge separation under deformation. Instead, a DE transducer has to be electrically pre-charged so that a current flow or change of voltage can be detected when it is deformed. Furthermore, the restricted observability for *q*_*p*_ = 0 is not only a drawback of the proposed approach. All referenced self-sensing methods have the same issue, but the usually superimposed voltage excitations ensure that this operating point does not occur. As this superimposed excitation is not required for the EKF-based estimator a certain amount of charge *q*_p,min_ is always applied, here.

**Figure 3:**
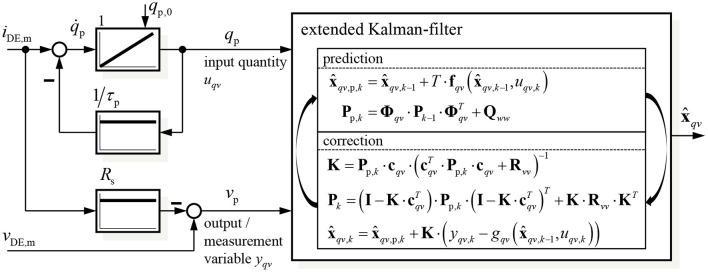
Structure of the proposed self-sensing state and disturbance estimator based on an extended Kalman-filter.

This results in the structure of the EKF-based self-sensing state and disturbance estimator shown in Figure 3. As the EKF will be implemented on a DSP its discrete implementation according to Welch and Bishop (2001) is applied. Using the external estimation of the charge by filtering the measured current *i*_DE,m_ has the advantage, that the state vector **x**_*qv*_ only includes mechanical states that have to be estimated with the EKF. Furthermore, the parameterization effort increases significantly with increasing system order so that it is meaningful to use a system with order *n* = 4 instead of *n* = 5.

For the implementation of the Kalman-filter algorithm according to Figure 3 the system (13) has to be linearized in the predicted state ${\hat{x}}_{qv,\text{p},k}$:

(16) $$\begin{array}{l} \;A_{qv,k}={\frac{\partial f_{qv}\left(x_{qv},u_{qv}\right)}{\partial x_{qv}}|}_{{\hat{x}}_{qv,\text{p},k},u_{qv,k}}\\ \;=\left[\begin{array}{cccc} 0 & 1 & 0 & 0\\ a_{qv,21} & \frac{-\gamma_{1}\cdot \eta_{\text{E}}}{1-{\hat{\varepsilon }}_{z,\text{p}}} & \frac{-\gamma_{1}\cdot E_{1}}{1-{\hat{\varepsilon }}_{z,\text{p}}} & \frac{-\gamma_{1}}{1-{\hat{\varepsilon }}_{z,\text{p}}}\\ 0 & 1 & -\frac{E_{1}}{\eta_{1}} & 0\\ 0 & 0 & 0 & 0 \end{array}\right],\\ \text{with }a_{qv,21}=\frac{\gamma_{1}}{1-{\hat{\varepsilon }}_{z,\text{p}}}\;\cdot \;\left[\frac{{\hat{\sigma }}_{\text{act},\text{p}}\left(u_{qv,k}\right)-{\hat{\sigma }}_{\text{load},\text{p}}}{1-{\hat{\varepsilon }}_{z,\text{p}}}-\frac{d{\hat{\sigma }}_{\text{elast},\text{p}}}{d{\hat{\varepsilon }}_{z,\text{p}}}-\frac{d{\hat{\sigma }}_{\text{el},\text{p}}}{d{\hat{\varepsilon }}_{z,\text{p}}}\right],\\ \text{and }\frac{d{\hat{\sigma }}_{\text{el},\text{p}}}{d{\hat{\varepsilon }}_{z,\text{p}}}\overset{\left(11\right)}{\underset{\left(7\right)}{=}}\;-\;\frac{\kappa \;\cdot \;q_{\text{p}}^{2}\;\cdot \;{\left(1-{\hat{\varepsilon }}_{z,\text{p}}\right)}^{\kappa -1}}{V\;\cdot \;C_{\text{p},0}}. \end{array}$$

Based on this the discrete transition matrix **Φ**_*qv*_ can be approximated by (Ifeachor and Jervis, 2002):

(17) $$\Phi_{qv}\approx I+A_{qv,k}\;\cdot \;T,$$

where *I* represents the unity matrix of order *n* = 4. The output vector $c_{qv,k}^{T}$ is calculated by the jacobian of the output function *g*_*qv*_ in Equation (13) with respect to the state vector **x**_*qv*_:

(18) $$\begin{array}{l} c_{qv,k}^{T}={\frac{\partial g_{qv}\left(x_{qv},\;u_{qv}\right)}{\partial x_{qv}}|}_{{\hat{x}}_{qv,rmp,k},u_{qv,k}}\\ \;=\left[-\frac{\kappa \;\cdot \;{\left(1-{\hat{\varepsilon }}_{z,p}\right)}^{\kappa -1}}{C_{\text{p},\text{0}}}\;\cdot \;u_{qv,k}\;0\;0\;0\right]. \end{array}$$

With these information the predicted state ${\hat{\text{x}}}_{qv,\text{p},k}$ and the related covariance matrix **P**_p,*k*_ can be determined in the prediction step (denoted by the index *p*) of the algorithm shown in Figure 3. In the following correction the Kalman matrix *K* and covariance matrix **P**_*k*_ are calculated to update the estimated state vector ${\hat{\text{x}}}_{qv,k}$. The covariance matrices of the measurement and system noise **R**_*vv*_ and **Q**_*ww*_, respectively, will be parameterized in the validation section 5. With the information, included in the state vector **x**_*qv,k*_ and Equation (4) to calculate the energy *U*_c,diel_ based on the charge *q*_p_, all state variables in **x** from Equation (6) as well as the load σ_load_ can be determined.

## 4. Self-Sensing Sliding Mode Control

**Figure 4:**
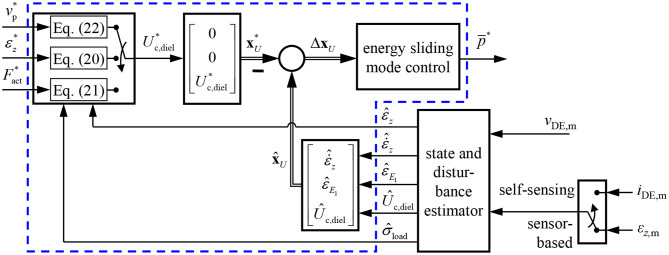
Detailed structure of the controller (blue box in Figure 1) including a feed-forward structure to either control the voltage, strain or force and a three-point controller with hysteresis and adaption of the inner flyback converter control.

The considered control plant modeled with Equation (6) has a strongly non-linear behavior. Furthermore, the bidirectional flyback converter allows to supply discrete feeding powers $\bar{p}$ so that it can be described by the three-point switch in Equation (5). Due to these properties the design of a variable structure control is well suited. In Hoffstadt and Maas (2017) a position controller based on the model (6) was introduced that uses the sliding mode control (SMC), for this purpose. Additionally, a SMC force controller was published in Hoffstadt and Maas (2018b). In the following it will be shown that this controller cannot be used to solely control the force *F*_act_ of the DE transducer but also the strain ε_*z*_ and the voltage *v*_p_ by applying different feed-forward structures to one and the same controller. This flexibility makes the approach advantageous for sophisticated applications like in soft robotics. The detailed design of the controller shown in Figure 4 can be found in Hoffstadt and Maas (2018b) and will be summarized in the following.

In case of the SMC a static setpoint state vector x^*^ has to be defined including setpoints for every state variable. Under consideration of the static force equilibrium

(19) $$\underset{t\to \infty }{\lim}\sigma_{\text{act}}\left(t\right)=\beta \;\cdot \;\sigma_{\text{el}}-\sigma_{\text{elast}}\overset{\left(4\right)}{=}\frac{2}{V}\;\cdot \;U_{\text{c},\text{diel}}-\sigma_{\text{elast}}\overset{!}{=}\sigma_{\text{load}}$$

resulting from Equation (2) setpoints for the energy $U_{\text{c},\text{diel}}^{*}$ can be derived. On the one hand, the energy can be calculated depending on a setpoint strain $\varepsilon_{z}^{*}$:

(20) $$U_{\text{c},\text{diel}}^{*}\left(\varepsilon_{z}^{*}\right)=\frac{V}{2}\;\cdot \;\left(\sigma_{\text{elast}}\left(\varepsilon_{z}^{*}\right)+\sigma_{\text{load}}\right).$$

To achieve this strain the electrostatic pressure caused by the energy according to Equation (4) has to compensate the elastic material tension $\sigma_{\text{elast}}\left(\varepsilon_{z}^{*}\right)$ given by Equation (3) as well as the influence of the disturbance σ_load_. On the other hand, if the DE transducer should generate a certain force $F_{\text{act}}^{*}=A\cdot \sigma_{\text{act}}$ the corresponding energy $U_{\text{c},\text{diel}}^{*}$ is given by:

(21) $$\begin{array}{l} U_{\text{c},\text{diel}}^{*}\;\left(F_{\text{act}}^{*}\right)=\frac{V}{2}\;\cdot \;\left(\frac{F_{\text{act}}^{*}}{A\left(\varepsilon_{z}\right)}+\sigma_{\text{elast}}\left(\varepsilon_{z}\right)\right),\\ \text{ with }\sigma_{\text{load}}=\frac{F_{\text{act}}^{*}}{A\left(\varepsilon_{z}\right)}\text{and }A\left(\varepsilon_{z}\right)=\frac{A_{0}}{1-\varepsilon_{z}}. \end{array}$$

In this case, the influence of the elastic deformation has to be compensated, i.e., the energy has to be increased with increasing strain ε_*z*_ (see Figure 2B).

Beside these approaches, the energy $U_{\text{c},\text{diel}}^{*}$ can be also determined depending on a setpoint voltage $v_{\text{p}}^{*}$ across the capacitance *C*_p_(ε_*z*_) in Equation (7):

(22) $$U_{\text{c},\text{diel}}^{*}\left(v_{\text{p}}^{*}\right)=\frac{1}{2}\;\cdot \;C_{\text{p}}\left(\varepsilon_{z}\right)\;\cdot \;v_{\text{p}}^{*}{}^{2}.$$

With Equations (20)–(22) three approaches exist to define a setpoint value for the energy $U_{\text{c},\text{diel}}^{*}$. For the system (6) also a setpoint for the strain $\varepsilon_{z}^{*}$ is required, while the other two state variables are zero during steady state ${\dot{\varepsilon }}_{z}=\varepsilon_{E_{1}}=0$, respectively. However, especially if the force or voltage should be controlled by applying Equations (21) or (22), they should be independent of the strain, i.e., that no setpoint $\varepsilon_{z}^{*}$ can be defined in this case. To overcome this issue, the control design is based on a reduced system (23) with ${\dot{\varepsilon }}_{z}$, ε_*E*_1__ and *U*_c,diel_ as state variables while the strain ε_*z*_ together with the load tension σ_load_ is considered to be a disturbance, here:

(23) $$\begin{array}{l} {\dot{x}}_{U}=\left[\begin{array}{c} {\ddot{\varepsilon }}_{z}\\ {\dot{\varepsilon }}_{E_{1}}\\ {\dot{U}}_{\text{c},\text{diel}} \end{array}\right]=\underset{f_{U}\left(x_{U},z\right)}{\underset{︸}{\left[\begin{array}{c} \frac{V}{m_{\text{B}}\;\cdot \;z_{0}^{2}}\;\cdot \;\frac{\sigma_{\text{act}}-\sigma_{\text{load}}}{1-\varepsilon_{z}}\\ {\dot{\varepsilon }}_{z}-\frac{E_{1}}{\eta_{1}}\;\cdot \;\varepsilon_{E_{1}}\\ -2\;\cdot \;U_{\text{c},\text{diel}}\;\cdot \;\left(\frac{{\dot{\varepsilon }}_{z}}{1-\varepsilon_{z}}+\frac{1}{\tau_{\text{p}}}\right) \end{array}\right]}}+\underset{b_{U}}{\underset{︸}{\left[\begin{array}{c} 0\\ 0\\ 1 \end{array}\right]}}\;\cdot \;{\bar{p}}^{\prime },\\ \text{with }z=\left[\begin{array}{c} \varepsilon_{z}\\ \sigma_{\text{load}} \end{array}\right]\cdot \end{array}$$

In this case, the setpoint state vector reads as:

(24) $${\dot{x}}_{U}^{*}={\left[\begin{array}{ccc} 0 & 0 & U_{\text{c},\text{diel}}^{*} \end{array}\right]}^{T}.$$

### 4.1. Design of the Sliding Mode

The control operation with a SMC is characterized by two phases. During the sliding mode the system is led toward its setpoint x^*^ on the switching function *S*(Δ**x**) = *S*(**x** − x^*^) = 0. Within the reaching phase it is ensured, first, that this switching function is reached from any arbitrary initial state. According to DeCarlo et al. (1988) one comparable simple approach for the design of the switching function is obtained if the system is in standard canonical form (denoted by the index *R*). To determine a corresponding transformation matrix *T*, the system (23) has to be linearized yielding the system matrix *A*_*U*_ for the estimated state ${\hat{\text{x}}}_{U}$:

(25) $$A_{U}={\frac{\partial f_{U}\left(x_{U},z\right)}{\partial x_{U}}|}_{x_{U}={\hat{x}}_{U}}=\left[\begin{array}{ccc} \frac{-\gamma_{1}\;\cdot \;\eta_{\text{E}}}{1-{\hat{\varepsilon }}_{z}} & \frac{-\gamma_{1}\;\cdot \;E_{1}}{1-{\hat{\varepsilon }}_{z}} & \frac{\gamma_{1}\;\cdot \;2/V}{1-{\hat{\varepsilon }}_{z}}\\ 1 & -\frac{E_{1}}{\eta_{1}} & 0\\ -\frac{2\;\cdot \;{\hat{U}}_{\text{c},\text{diel}}}{1-{\hat{\varepsilon }}_{z}} & 0 & -\frac{2\;\cdot \;{\hat{\dot{\varepsilon }}}_{z}}{1-{\hat{\varepsilon }}_{z}}-\frac{2}{\tau_{\text{p}}} \end{array}\right].$$

As the system behaves linear concerning the input *u*, the constant input vector **b**_*U*_ is already given in Equation (23). With these information the following transformation matrix *T* can be derived as proposed by Kalman (1960):

(26) $$\begin{array}{l} T=-\frac{V}{2}\;\cdot \;\frac{1-{\hat{\varepsilon }}_{z}}{\gamma_{1}}\;\cdot \;\left[\begin{array}{ccc} 0 & -1 & 0\\ -1 & \frac{E_{1}}{\eta_{1}} & 0\\ t_{31} & t_{32} & -\frac{2}{V}\;\cdot \;\frac{\gamma_{1}}{1-{\hat{\varepsilon }}_{z}} \end{array}\right]\\ \text{with }t_{31}=\frac{E_{1}}{\eta_{1}}+\frac{\gamma_{1}\;\cdot \;\eta_{E}}{1-{\hat{\varepsilon }}_{z}}\text{ and }t_{32}=\frac{E_{1}}{\eta_{1}}\;\cdot \;\left(\frac{\gamma_{1}\;\cdot \;\eta_{1}}{1-{\hat{\varepsilon }}_{z}}-\frac{E_{1}}{\eta_{1}}\right). \end{array}$$

For the considered single input single output (SISO) system a linear switching function is defined:

(27) $$\begin{array}{l} S\left(\Delta x_{\text{R}}\right)=c^{T}\;\cdot \;\Delta x_{\text{R},U}=\left[\begin{array}{ccc} c_{1} & c_{2} & c_{3} \end{array}\right]\;\cdot \;T\;\cdot \;\left({\hat{x}}_{U}-x_{U}^{*}\right),\\ \text{ with }\Delta x_{\text{R},U}=T\;\cdot \;\left({\hat{x}}_{U}-x_{U}^{*}\right). \end{array}$$

During the sliding mode *S*(Δ*x*_R_) = 0 as well as Ṡ(Δ*x*_R_) = 0 applies. This behavior is obtained by the equivalent input (DeCarlo et al., 1988)

(28) $$\begin{array}{l} u_{\text{eq}}\;=-{\left(c^{T}\;\cdot \;b_{\text{R},U}\right)}^{-1}\;\cdot \;c^{T}\;\cdot \;A_{\text{R},U}\;\cdot \;\Delta x_{\text{R},U},\\ \text{ with }A_{\text{R},U}=T\;\cdot \;A_{U}\;\cdot \;T^{-1}\text{ and }b_{\text{R},U}=T\;\cdot \;b_{U}. \end{array}$$

With this input the dynamics during the sliding mode only depend on the coefficients *c*_*i*_, with *i* = 1, 2, 3, of the switching function in Equation (27):

(29) $$\begin{array}{l} \Delta {\dot{x}}_{\text{R},U}=A_{\text{R},U}\;\cdot \;\Delta x_{\text{R},U}+b_{\text{R},U}\;\cdot \;u_{\text{eq}}\\ \;=\left[I-b\;\cdot \;{\left[c^{T}\;\cdot \;b\right]}^{-1}\;\cdot \;c^{T}\right]\;\cdot \;A_{\text{R},U}\;\cdot \;\Delta x_{\text{R},U}\\ \;=\left[\begin{array}{ccc} 0 & 0 & 0\\ 0 & 0 & 1\\ 0 & -\frac{c_{1}}{c_{3}} & -\frac{c_{2}}{c_{3}} \end{array}\right]\;\cdot \;\Delta x_{\text{R}}=\left[\begin{array}{cc} 0 & 0^{T}\\ 0 & {\tilde{A}}_{1} \end{array}\right]\;\cdot \;\Delta x_{\text{R},U}.\; \end{array}$$

An other characteristic property of the SMC approach is that during the sliding mode the system order *n* is reduced by the number of inputs *p* (here *p* = 1). Thus, the dynamics during the sliding mode can be defined by a pole placement under consideration of ${\tilde{A}}_{1}$. For a second order element with damping coefficient *D* and cut-off frequency ω_g_ this results in:

(30) $$\begin{array}{l} \det\left(s\;\cdot \;I-{\tilde{A}}_{1,U}\right)=s^{2}+\frac{c_{2}}{c_{3}}\;\cdot \;s+\frac{c_{1}}{c_{3}}\overset{!}{=}\;s^{2}+2\;\cdot \;D\;\cdot \;\omega_{\text{g}}\;\cdot \;s+\omega_{\text{g}}^{2}\\ \text{ with }c_{3}=1,\;\Rightarrow \;c_{1}=\omega_{\text{g}}^{2},\;c_{2}=2\;\cdot \;D\;\cdot \;\omega_{\text{g}}. \end{array}$$

### 4.2. Reachability

To reach this sliding mode a proper controller function *u*(Δ*x*_R,*U*_) has to be determined and parametrized under consideration of the properties of the feeding power electronics. One approach to prove the reachability is based on an investigation of the Laypunov function $V\left(\Delta x_{\text{R},U}\right)=1/2\cdot S^{2}\left(\Delta x_{\text{R},U}\right)$. To ensure stable steady-state behavior the time derivate of the Lyapunov function has to be negative:

(31) $$\dot{V}\left(\Delta x_{\text{R},U}\right)=S\left(\Delta x_{\text{R},U}\right)\;\cdot \;\dot{S}\left(\Delta x_{\text{R},U}\right)\overset{!}{<}0.$$

The derivative of the switching function is given by:

(32) $$\begin{array}{l} \dot{S}\left(\Delta x_{\text{R},U}\right)=c^{T}\;\cdot \;T\;\cdot \;\left(A_{U}\;\cdot \;\Delta x_{U}+b_{U}\;\cdot \;u\left(\Delta x_{\text{R},U}\right)\right)\\ \;=\zeta_{1}\;\cdot \;\Delta {\dot{\varepsilon }}_{z}+\zeta_{2}\;\cdot \;\Delta \varepsilon_{E1}+\zeta_{3}\;\cdot \;\Delta U_{\text{c},\text{diel}}+u\left(\Delta x_{\text{R},U}\right),\text{ with} \end{array}$$

(33a) $$\begin{array}{l} \zeta_{1}=\frac{V}{2\;\cdot \;\gamma_{1}}(\omega_{\text{g}}^{2}-2\;\cdot \;D\;\cdot \;\omega_{\text{g}}\;\cdot \;\left(\gamma_{1}\;\cdot \;\eta_{\text{E}}+\frac{E_{1}}{\eta_{1}}\right)\\ \;+\gamma_{1}^{2}\;\cdot \;\eta_{\text{E}}^{2}+\gamma_{1}\;\cdot \;E_{1}\;\cdot \;\left(\frac{\eta_{\text{E}}}{\eta_{1}}-1\right)+\frac{E_{1}^{2}}{\eta_{1}^{2}}), \end{array}$$

(33b) $$\begin{array}{l} \zeta_{2}=\frac{V\;\cdot \;E_{1}}{2\;\cdot \;\gamma_{1}\;\cdot \;\eta_{1}}\;\cdot \;(\omega_{\text{g}}^{2}+2\;\cdot \;D\;\cdot \;\omega_{\text{g}}\;\cdot \;\left(\gamma_{1}\;\cdot \;\eta_{1}-\frac{E_{1}}{\eta_{1}}\right)\\ \;-2\;\cdot \;\gamma_{1}\;\cdot \;E_{1}-\gamma_{1}^{2}\;\cdot \;\eta_{\text{E}}\;\cdot \;\eta_{1}+\frac{E_{1}^{2}}{\eta_{1}^{2}})\text{ and} \end{array}$$

(33c) $$\zeta_{3}=2\;\cdot \;D\;\cdot \;\omega_{\text{g}}-\frac{2}{\tau_{\text{p}}}-\frac{E_{1}}{\eta_{1}}-\gamma_{1}\;\cdot \;\eta_{\text{E}},\text{ for }{\hat{x}}_{U}=0.$$

The coefficients ζ_1_, ζ_2_ and ζ_3_ depend on material parameters as well as the damping ratio *D* and cut-off frequency ω_g_. These two controller parameters are chosen in such a way that the influence of the state variables $\Delta {\dot{\varepsilon }}_{z}$ and Δε_*E*_1__ on Equation (32) vanishes. By solving ζ_1_ = 0 and ζ_2_ = 0 the following parameters result:

(34a) $$\omega_{\text{g},0}=\sqrt{\gamma_{1}\cdot E_{1}\cdot \frac{\eta_{1}+\eta_{\text{E}}}{\eta_{1}}}\text{and}$$

(34b) $$D_{0}=\frac{1}{2}\cdot \sqrt{\frac{{\left(E_{1}+\gamma_{1}\cdot \eta_{1}\cdot \eta_{\text{E}}\right)}^{2}}{\gamma_{1}\cdot E_{1}\cdot \eta_{1}\cdot \left(\eta_{1}+\eta_{\text{E}}\right)}}.$$

According to Equation (5) the input power $\bar{p}$ supplied by the bidirectional flyback converter can be described by a three-point controller. However, for the design of the SMC the off-state with $\bar{p}=0$ can be neglected in a first step. Under consideration $\bar{p}=\pm {\bar{p}}_{\max}$ a two-point controller is defined:

(35) $$u\left(\Delta x_{\text{R},U}\right)=\text{sgn}\left(S\left(\Delta x_{\text{R},U}\right)\right)\cdot \varrho .$$

The parameter $\varrho =\pm {\bar{p}}_{\max}$ will be chosen so that the reachability is ensured.

By inserting Equations (34), (34b), and (35) into Equation (32) the time derivative of the switching function simplifies to:

(36) $$\begin{array}{l} \dot{S}\left(\Delta x_{\text{R},U}\right)=-\frac{2}{\tau_{\text{p}}}\;\cdot \;\Delta U_{\text{c},\text{diel}}+u\left(\Delta x_{\text{R},U}\right)\\ \;=\frac{2}{\tau_{\text{p}}}\;\cdot \;U_{\text{c},\text{diel}}^{*}+\text{sgn}\left(S\left(\Delta x_{\text{R},\varepsilon }\right)\right)\;\cdot \;\varrho . \end{array}$$

The control parameter ϱ is determined by applying a case-by-case analysis to satisfy Equation (31):

(37) $$\begin{array}{l} \text{I.: }S\;\left(\Delta x_{\text{R},U}\right)\;>\;0,\;\Rightarrow \;u\;\left(\Delta x_{\text{R},U}\right)\;=\;+\varrho ,\;\\ \;\Rightarrow \dot{S}\;\left(\Delta x_{\text{R},U}\right)\;=\frac{2}{\tau_{\text{p}}}\;\cdot \;U_{\text{c},\text{diel}}^{*}+\varrho \overset{!}{<}\;0,\\ \text{II.: }S\left(\Delta x_{\text{R},U}\right)\;<\;0,\;\Rightarrow u\left(\Delta x_{\text{R},U}\right)\;=-\varrho ,\\ \;\Rightarrow \dot{S}\left(\Delta x_{\text{R},U}\right)\;=\frac{2}{\tau_{\text{p}}}\;\cdot \;U_{\text{c},\text{diel}}^{*}-\varrho \overset{!}{>}0. \end{array}$$

As the energy $U_{\text{c},\text{diel}}^{*}$ will always be equal to or larger than zero, both inequalities are solved by choosing:

(38) $$\varrho =-{{\bar{p}}^{\prime }}_{\max}\;\Rightarrow \;{{\bar{p}}^{\prime }}_{\max}>\frac{2}{\tau_{\text{p}}}\;\cdot \;U_{\text{c},\text{diel}}^{*}.$$

Especially during steady state the introduced two-point controller will permanently switch between the positive and negative input power $\pm {\bar{p}}_{\max}$. To avoid this chattering, the controller is extended to a three-point controller with hysteresis, as already shown in Figure 4:

(39) $${\bar{p}}^{*}=\{\begin{array}{cc} +{\bar{p}}_{\max}, & \begin{array}{c} \text{for }S\left(\Delta x_{\text{R},U}\right)\leq -\delta_{S}\text{ or}-\delta_{S}<S\left(\Delta x_{\text{R},U}\right)<0\wedge \dot{S}\left(\Delta x_{\text{R},U}\right)>0 \end{array}\\ 0, & \text{else}\\ -{\bar{p}}_{\max} & \begin{array}{c} \text{for }S\left(\Delta x_{\text{R},U}\right)\geq \delta_{S}\text{ or}0<S\left(\Delta x_{\text{R},U}\right)<\delta_{S}\wedge \dot{S}\left(\Delta x_{\text{R},U}\right)<0 \end{array} \end{array}.$$

On the one hand, the off-state of the flyback converter is now taken into account, while on the other hand the hysteresis with threshold δ_*S*_ will significantly reduce the switching frequency in closed-loop operation. In Figure 4 an output limitation is also depicted that switches off the control, when the energy Û_c,diel_ exceeds a maximum value *U*_c,diel,max_. Furthermore, to improve the steady state behavior the inner control of the flyback converter is adapted. Depending on the absolute value of the switching function |*S*(Δ**x**_R,*U*_)|, the maximum magnetizing current $I_{m,\max}^{*}$ and thus the feeding power $\bar{p}$ according to Equation (5) is varied. This ensures, that for large control deviations corresponding to large values of |*S*(Δ**x**_R,*U*_)| the maximum feeding power is supplied for achieving the maximum dynamics. In contrast, for small control deviations the power is reduced for a higher accuracy by also adapting the hysteresis threshold δ_*S*_. Further details can be found in Hoffstadt and Maas (2017, 2018b).

## 5. Experimental Validation

### 5.1. Test Setup for the Experimental Validation

**Figure 5:**
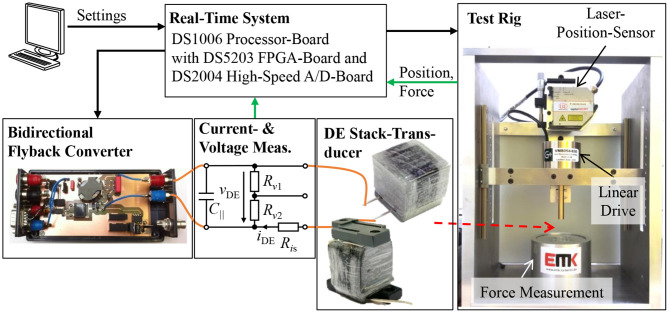
Test setup for the experimental validation comprising a bidirectional flyback converter, a voltage and current measurement, a DE stack-transducer and a test rig with linear drive, while the data logging and the different controls are implemented on the real-time system.

Figure 5 schematically depicts the test setup used for the experimental validation of the self-sensing estimator and the self-sensing control. It consists of a bidirectional flyback converter that supplies the DE transducer with voltages up to 2.5 kV (Hoffstadt and Maas, 2016). The voltage *v*_DE,m_ is measured with the voltage probe TT-SI 9010 from Testec, while the current *i*_DE,m_ is determined by the voltage drop across the shunt resistance *R*_*is*_ = 1 kΩ. Details about the utilized DE transducers can be found in Maas et al. (2015). If no-load scenarios are investigated in the following, the displacement of the DE transducer is directly measured with the laser sensor OptoNCDT ILD 2300 from Micro-Epsilon. To apply loads to the DE transducer the test rig on the right hand side of Figure 5 will be used. It consists of a force measurement with the force sensor 9217A from Kistler and a voice coil linear drive VM8054-630 from Geeplus. The DE transducer can be attached between the force measurement and the linear drive. Via the voice coil load profiles with high dynamics can be applied to the DE transducer, while the resulting actuator force *F*_act,m_ is measured. Here, the same laser sensor as for the no-load scenarios measures the displacement of the rigidly coupled voice coil and DE transducer.

The proposed self-sensing algorithm and the energy control are implemented on the DSP of a real-time system from dSPACE operating with a sample rate of *f*_DSP_ = 20 kHz. The system contains also a fast FPGA board. On this board the control of the flyback converter and the signal conditioning for the measured voltage and current *v*_DE,m_ and *i*_DE,m_ are performed.

### 5.2. Validation of the EKF-Based Self-Sensing Algorithm

Before the closed-loop self-sensing operation is investigated, the estimation results obtained with the suggested self-sensing approach are compared to results estimated with the sensor-based observer introduced in Hoffstadt and Maas (2017, 2018b). The parameters of the silicone based DE stack-transducer with *N* = 192 layers are listed in Table 1. This table also includes parameters for the controller used in the following section.

**Table 1:** Parameters of the utilized silicone based DE stack-transducer and the self-sensing controller.

***Y***	**η_*E*_**	***E*_1_**	**η_1_**	***V***	***m*_*B*_**	***N*·*d*_0_ = *z*_0_**	**τ_*p*_**
1.08 MPa	490 Pa·s	155 kPa	1.7 kPa·s	1.4 cm^3^	0.5 g	9.6 mm	24 s
*C*_p,0_	*R*_*s*_	*R*_*vv*_	ω_g_	*D*	*I*_min_	*I*_max_	δ_*S*_
6 nF	135 kΩ	4 V^2^	2.430 rad/s	3	4 A	8 A	4·Δ*U*_max_

**Figure 6:**
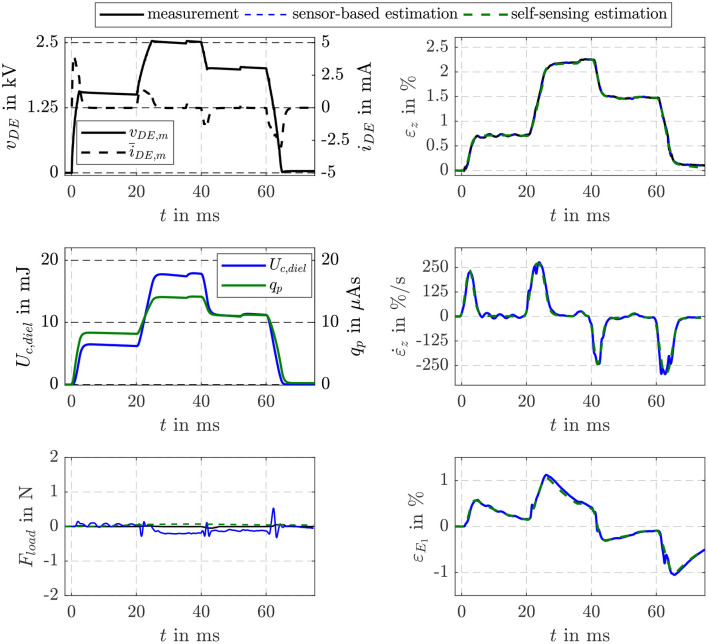
Comparison of the EKF-based estimation results of the proposed self-sensing filter with a sensor-based filter for the no-load scenario.

Figure 6 compares the estimation results of the proposed self-sensing approach with the sensor-based estimator. The voltage controlled bidirectional flyback converter supplies the DE stack-transducer stepwise with voltages of *v*_DE_ = 1.5, 2.5, and 2 kV, respectively. The charge *q*_p_ determined by filtering the measured current *i*_DE,m_ according to Equation (10) is used as input for the self-sensing filter, while the sensor-based estimator uses the energy *U*_c,diel_ as input.

The measurement noise *R*_*vv*_ = 4 V^2^ required for the implementation of the EKF can be determined experimentally. For this, the output function *g*_*qv*_ in Equation (13) and the properties of the voltage probe and current measurement via the shunt have to be taken into account. One of the main issues when designing an EKF is to find an appropriate choice of **Q**_*ww*_. Here, the numerical optimization approach presented by Powell (2002) is used to minimize the error between simulated and estimated state variables by varying the entries of the symmetric matrix **Q**_*ww*_. For the system introduced in section 3 this optimization yields:

(40) $$\begin{array}{l} Q_{ww,qv}\\ =\zeta_{qv}\;\cdot \;\left[\begin{array}{cccc} 4,8\cdot 10^{-8} & -1,7\cdot 10^{-9} & -2,2\cdot 10^{-8} & -9,3\cdot 10^{-4}\\ -1,7\cdot 10^{-9} & 4,9\cdot 10^{-5} & 1,5\cdot 10^{-4} & 4,4\cdot 10^{-1}\\ -2,2\cdot 10^{-8} & 1,5\cdot 10^{-4} & 1,6\cdot 10^{-9} & -1,7\cdot 10^{-5}\\ -9,3\cdot 10^{-4} & 4,4\cdot 10^{-1} & -1,7\cdot 10^{-5} & 7,6\cdot 10^{4} \end{array}\right]. \end{array}$$

The entries represent in a certain way the uncertainty of the model (13) to describe the dynamics of the state variables. While all entries of the matrix are comparable small, the one in the fourth row and column is very large. This is due to the unknown dynamics of the load tension that is considered with ${\dot{\sigma }}_{\text{load}}=0$ in Equation (13). As the dynamics of the state estimation can be adjusted by the absolute values of the entries in **Q**_*ww*_ the scaling factor ζ_*qv*_ is introduced. It gives the opportunity to adjust a compromise between sufficient dynamics, reliable state estimation and noise suppression.

In Figure 6 the no-load scenario with *F*_load_ = *A* · σ_load_ = 0 is considered. As can be seen in the comparison of the measured and estimated strains ε_*z*_ in the top right plot, almost no deviations between the approaches in terms of dynamics and accuracy occur. Due to parameter deviations the sensor-based filter estimates small load forces especially during transient operation. For the self-sensing filter with $\zeta_{qv}=10^{-3}$ a comparable small factor is applied here. With this negligible deviations in the estimated load force occur without affecting the estimation results of the state variables shown on the right.

**Figure 7:**
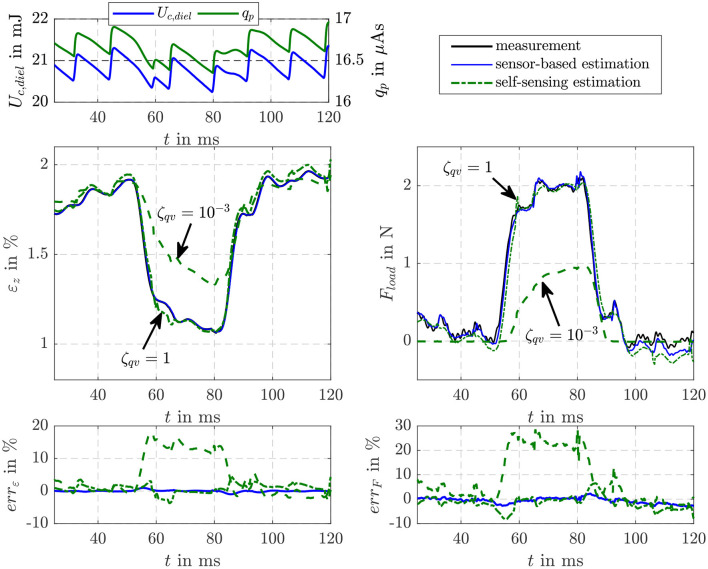
Comparison of the EKF-based estimation results of the proposed self-sensing filter with a sensor-based filter when a load force is applied to the DE transducer.

Figure 7 compares the estimation results obtained when a load force of *F*_load_ = 2 N is stepwise applied to the DE stack-transducer with the force controlled voice coil actuator. When the tensile load is applied the strain of the DE transducer reduces from ε_*z*_ ≈ 1.9% to ε_*z*_ ≈ 1.1%. In voltage controlled operation this causes a reduction of charge and energy as can be seen in the top left plot. The saw tooth profile in the charge *q*_p_ and energy *U*_c,diel_ is caused by the voltage control of the flyback converter that is based on a hysteresis controller. The sensor-based filter estimates the strain as well as the load force with errors less than |*err*_ε_| ≤ 1% and |*err*_*F*_| ≤ 4%, respectively. For the self-sensing filter two parameterization with $\zeta_{qv}=10^{-3}$ and ζ_*qv*_ = 1 are investigated. While with $\zeta_{qv}=10^{-3}$ the strain and force are estimated with errors below |*err*_ε_| ≤ 1% before the load is applied and after it is released, the dynamics of the estimation is not sufficient to consider the influence of the load correctly. In contrast, with ζ_*qv*_ = 1 the influence of the load is accurately estimated. However, with this setting the noise suppression especially for charge states below *q*_p_ ≤ 5 μAs is not sufficient. Therefore, for the following investigations of the self-sensing control the scaling factor is switched from $\zeta_{qv}=10^{-3}$ to ζ_*qv*_ = 1 if the charge exceed *q*_p_ ≤ 5 μAs. This ensures an accurate estimation of the inner transducer states at low charge states as well as an accurate detection of a load force and its influence on the states.

### 5.3. Validation of the Self-Sensing Control

**Figure 8:**
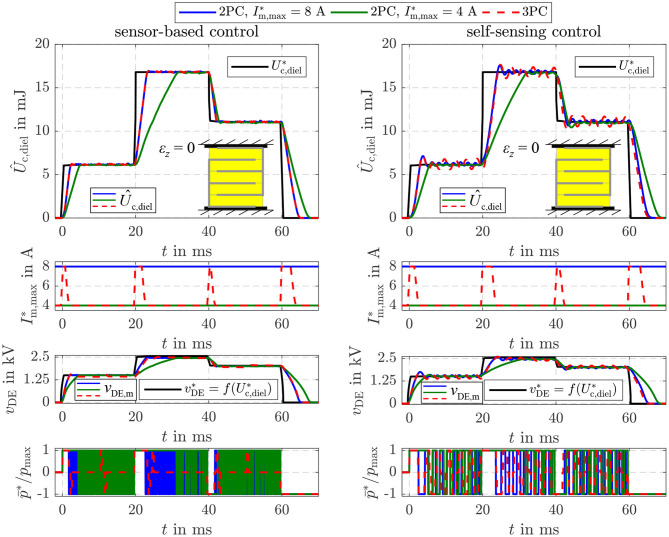
Comparison of the sensor-based and self-sensing sliding mode energy control. In both cases two-point controllers (2PC) with $I_{\text{m},\max}^{*}=8$ A and $I_{\text{m},\max}^{*}=4$ A as well as the three-point controller (3PC) with hysteresis and adaption of the inner flyback converter control are considered.

The parameters of the sliding mode energy controller designed in section 4 are listed in Table 1. The damping coefficient *D* = 3 and cut-off frequency ω_g_ = 2.430 rad/s were determined with Equation (34). The hysteresis threshold δ_*S*_ = 4 · Δ*U*_max_ for the three-point controller in Equation (39) is set to a multiple of the energy increment Δ*U*_max_ transfered during one switching period *T*_S_ of the flyback converter. Figure 8 compares the closed-loop operation of the sensor-based controller published in Hoffstadt and Maas (2018b) and the proposed self-sensing controller. First of all, no feed-forward control approaches as suggested in Equations (20)–(22) are considered. Instead, three setpoint steps for the energy $U_{\text{c},\text{diel}}^{*}$ are applied that correspond to voltages of *v*_DE_ = 1.5 kV, 2.5 kV and 2 kV for the silicone based DE-stack-transducer, respectively. For both, the sensor-based and the self-sensing control two-point controllers (2PC) according to Equation (35) with $I_{\text{m},\max}^{*}=8$ A and $I_{\text{m},\max}^{*}=4$ A are investigated as well as the three-point controller (3PC) with hysteresis and adaption of the inner flyback converter control from Equation (39) and Figure 4. The DE stack-transducer is attached between the force measurement and the blocked voice coil so that it cannot deform (ε_*z*_ = 0) to avoid disturbances.

Via the setpoint $I_{\text{m},\max}^{*}$ for the current control of the flyback converter its feeding power is adjusted according to Equation (5). Due to the reduced power it takes a longer time to adjust the setpoint energies with the two-point controller with $I_{\text{m},\max}^{*}=4$ A compared to the one with $I_{\text{m},\max}^{*}=8$ A. In contrast, the reduced feeding power results in a higher accuracy during steady state. The standard deviation for the time interval between 50 and 60 ms increases from 0.03 mJ (2PC, $I_{\text{m},\max}^{*}=4$ A) to 0.05 mJ (2PC, $I_{\text{m},\max}^{*}=8$ A) for the sensor-based control and from 0.1 to 0.3 mJ for the self-sensing control, respectively. The adaptive three-point controller with hysteresis combines the advantageous of the two mentioned two-point controllers by automatically choosing the maximum current $I_{\text{m},\max}^{*}=8$ A right after setpoint steps and reducing this current to $I_{\text{m},\max}^{*}=4$ A at steady state. This fundamental behavior applies for both the sensor-based and self-sensing control. Although the dynamics of both approaches are comparable, a small oscillation around the setpoint can be observed in case of the self-sensing control that results in the higher standard deviation.

Furthermore, it can be seen that the two-point controllers permanently switch between the maximum charging and discharging power $\bar{p}=\pm {\bar{p}}_{\max}$ during steady state. By extending the controller to a three-point controller with hysteresis, the switching frequency can be significantly reduced by more than 80% in case of the sensor-based control and 30% in case of the self-sensing control.

**Figure 9:**
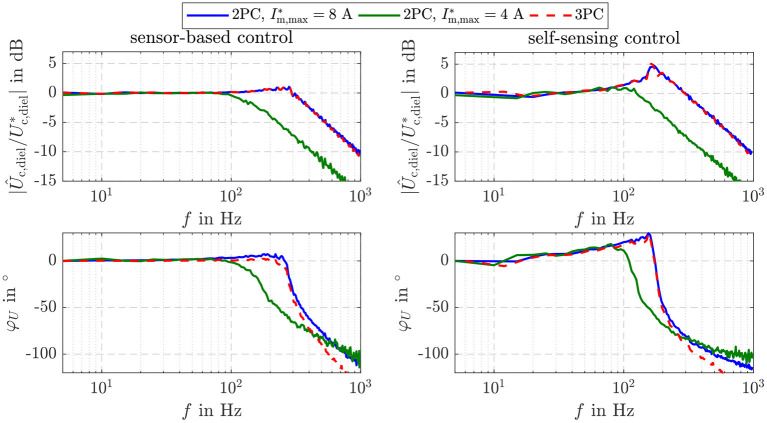
Bandwidth of the sensor-based and self-sensing sliding mode energy control for the three investigated controller settings.

Figure 9 depicts the comparison of the bandwidth of the introduced controller settings. For this purpose, the small signal behavior is considered. A harmonic setpoint $U_{\text{c},\text{diel}}^{*}$ with increasing frequency, an offset of Ū_c,diel_ = 12 mJ and an amplitude of *U*_c,diel,amp_ = 2 mJ is applied. The sensor-based two-point controller with $I_{\text{m},\max}^{*}=8$ A and the three-point controller have a high -3 dB cut-off frequency of about 400 Hz. This is also obtained with the self-sensing control. However, disruptive amplitude peaks of about 5 dB result in the already observed oscillation. By reducing the feeding power $\bar{p}$ with $I_{\text{m},\max}^{*}=4$ A, the cut-off frequency is reduced to 200 Hz, while the amplitude peaks are suppressed.

### 5.4. Energy Control With Voltage Feed-Forward Control

**Figure 10:**
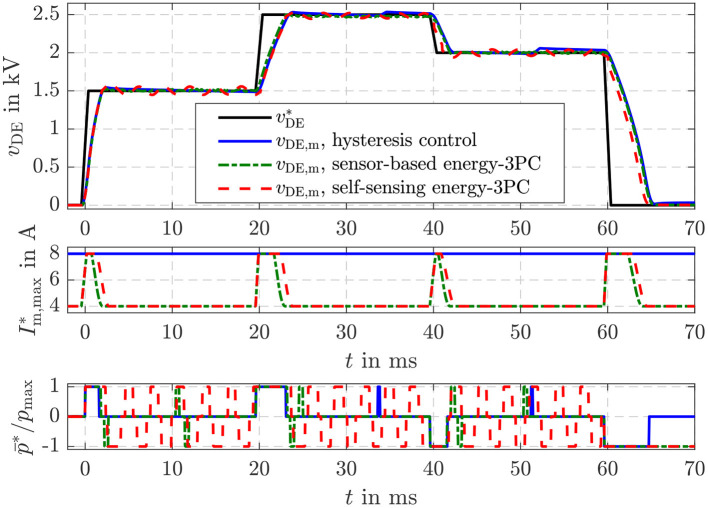
Comparison of a simple hysteresis voltage control with the sensor-based and self sensing energy three-point controller using a voltage feed-forward control.

By applying Equation (22) for the feed-forward control depicted in Figure 4 the voltage *v*_p_ across the capacitance *C*_p_ can be controlled. In Figure 10 the results of the sensor-based and self-sensing energy three-point controller are compared to the behavior obtained with the hysteresis voltage control for the bidirectional flyback converter suggested in Hoffstadt and Maas (2016).

For the hysteresis voltage control a threshold of Δ*v*_DE_ = 30 V was chosen. If the control deviation $\left|v_{\text{DE}}^{*}-v_{\text{DE},\text{m}}\right|$ exceeds this threshold the control activates the flyback converter to charge or discharge the DE transducer. Afterwards the converter is turned into idle state again. The three-point controller suggested here behaves more or less the same. The only difference is that with the controller settings from Table 1 a threshold of *v*_p_ ≈ 16 V results. This smaller threshold increases on the one hand the steady state accuracy. However, the switching frequency is on the other hand a bit higher compared to the simple hysteresis voltage control. Concerning the sensor-based and self-sensing energy control a comparable behavior as shown and explained in Figure 8 can be observed here, too.

### 5.5. Energy Control With Position Feed-Forward Control

**Figure 11:**
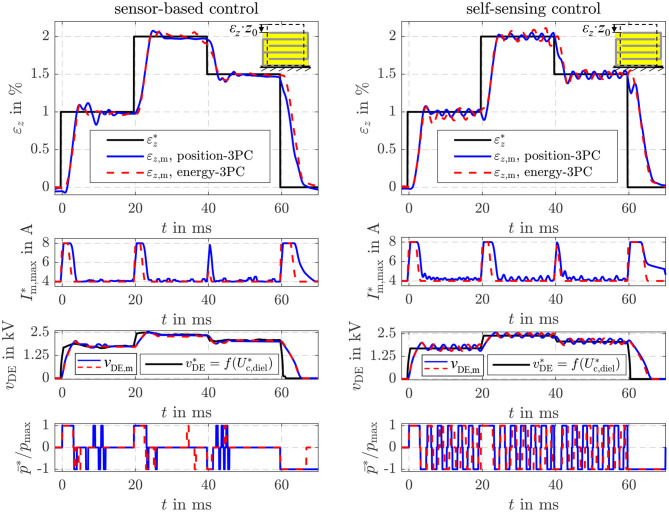
Comparison of the sensor-based and self-sensing energy control with position feed-forward control and an explicit sliding mode position control.

If the setpoint energy $U_{\text{c},\text{diel}}^{*}$ of the control structure in Figure 4 is determined with Equation (20) the proposed energy control can be used to adjust a certain strain $\varepsilon_{z}^{*}$, although the strain is not part of the state vector **x**_*U*_. To compensate the influence of a disturbance, the estimated load ${\hat{\sigma }}_{\text{load}}$ is considered in Equation (20). In contrast, an explicit position control based on the model (6) was derived in Hoffstadt and Maas (2017). Figure 11 shows the comparison of the explicit (Position-3PC) and energy-based position control (Energy-3PC) for the no-load case of the DE stack-transducer. Both approaches are realized as sensor-based and self-sensing control with the adaptive three-point controller from Equation (39).

The explicit and energy-based position control show comparable dynamics and accuracy for both the sensor-based and the self-sensing control. The different setpoints are adjusted within a few milliseconds. By increasing the strain setpoint $\varepsilon_{z}^{*}$ the energy $U_{\text{c},\text{diel}}^{*}$ also increases according to Equation (20). Instead of the energy, the voltage *v*_DE_ is depicted in Figure 11 as it is measured directly and can be interpreted more intuitively. With Equation (22) a relationship between the voltage *v*_p_ ≈ *v*_DE_ is given. As the no-load case is considered here, for a constant setpoint of the strain $\varepsilon_{z}^{*}$ a constant setpoint for the energy $U_{\text{c},\text{diel}}^{*}$ or the voltage results, respectively.

**Figure 12:**
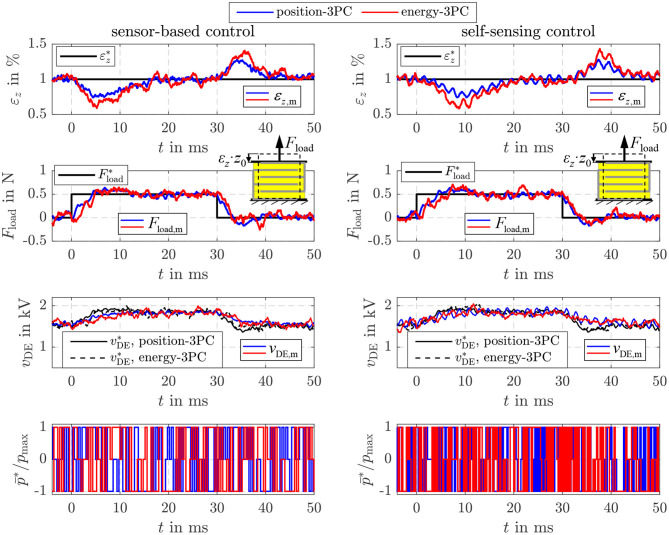
Disturbance reaction of the sensor-based and self-sensing energy control with position feed-forward control and an explicit sliding mode position control.

In addition, Figure 12 depicts the disturbance reaction of the different position control approaches. For this purpose, a tensile load force of $F_{\text{load}}^{*}=$ 0.5 N is applied by the linear drive of the test rig in Figure 5, while the setpoint strain is constantly set to $\varepsilon_{z}^{*}=$ 1%. Right after the load is applied, the strain deviates from its setpoint due to the influence of the disturbance. However, the load is estimated with the sensor-based as well as the self-sensing EKF. According to Equation (20) the setpoint energy $U_{\text{c},\text{diel}}^{*}$, or voltage $v_{\text{DE}}^{*}$, respectively, is increased to compensate the influence of the disturbance ${\hat{\sigma }}_{\text{load}}$. In Figure 12 this behavior can be seen in the response of the corresponding voltage *v*_DE_ in the third subplot. This compensates the influence of the disturbance within approx. 15 ms. In case of the energy-based position control a slightly higher control deviation can be observed after the load steps. This is mainly due to the fact, that the energy control only reacts on control deviations of the energy *U*_c,diel_, while the explicit position control considers the control deviation of the strain ε_*z*_ directly.

### 5.6. Energy Control With Force Feed-Forward Control

**Figure 13:**
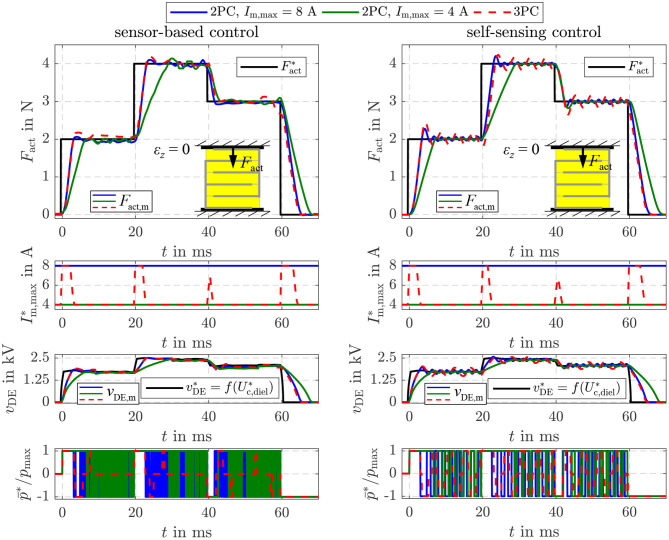
Comparison of the sensor-based and self-sensing sliding mode energy control with force feed-forward control. In both cases two-point controllers (2PC) with $I_{\text{m},\max}^{*}=8$ A and $I_{\text{m},\max}^{*}=4$ A as well as the three-point controller (3PC) with hysteresis and adaption of the inner flyback converter control are considered.

Beside the two validated approaches, Equation (21) offers the opportunity to realize a force feed-forward control under consideration of the current elastic material tension $\sigma_{\text{elast}}\left({\hat{\varepsilon }}_{z}\right)$ based on the proposed energy control as already depicted in Figure 4. As for the previous two approaches, the controller settings are the same as listed in Table 1. However, in Figure 13 also the two-point controller with *I*_m,max_ = 8 A and *I*_m,max_ = 4 A is considered again. The deformation of the DE stack-transducer is blocked in this case to investigate the control behavior caused by setpoint steps without any disturbance. In general, a comparable behavior to the pure energy control in Figure 8 can be observed here, too. According to Equation (21) a constant energy setpoint $U_{\text{c},\text{diel}}^{*}$ is obtained for a certain force $F_{\text{act}}^{*}$ and ε_*z*_ = 0. In comparison to the two-point controllers the adaptive three-point controller from Equation (39) ensures the highest possible dynamics by the maximum current *I*_m,max_ = 8 A during transient operation as well as good steady state accuracy with significantly reduced switching frequency by reducing the current to *I*_m,max_ = 4 A. The self-sensing control adjusts the different setpoint forces with dynamics that are absolutely comparable to the sensor-based control.

**Figure 14:**
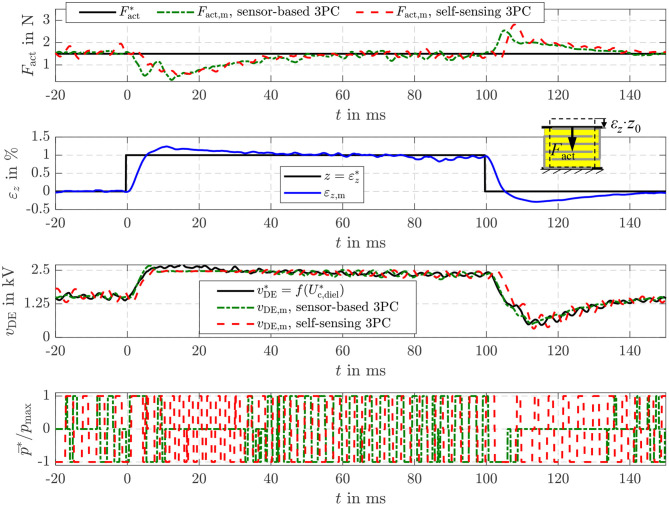
Disturbance reaction of the sensor-based and self-sensing energy control with force feed-forward control.

In Figure 14 also the disturbance reaction of the energy-based force control is shown. In this case a variable strain ε_*z*_ adjusted by the position-controlled linear drive acts as a disturbance. The stepwise change of the strain to $\varepsilon_{z}^{*}=$ 1% causes a reduction of the force *F*_act_ in the first moment. However, by increasing the setpoint energy $U_{\text{c},\text{diel}}^{*}$, or voltage $v_{\text{DE}}^{*}$, respectively, according to Equation (21) the influence is compensated comparable to the behavior observed for the disturbance reaction of the energy-based position control in Figure 12. The sensor-based control offers a marginal better control quality what is caused by the slightly higher dynamics of the sensor-based state and disturbance estimation.

The investigation of the pure energy control in Figures 8, 9 as well as of the different feed-forward controls in the Figures 10–14 proved that both high dynamics and good steady state accuracy are obtained with the proposed self-sensing control approach. The developed self-sensing EKF estimates not only the inner states of the DE transducer but also an external load tension. In case of the position feed-forward control this allows to compensate the influence of a disturbance load. In contrast, if the force feed-forward control is applied the elastic material tension caused by a deformation of the DE transducer is reliably compensated. Furthermore, extensions to an adaptive three-point controller enabled a reduction of the switching frequency of up to 80% to increase the energy efficiency without reducing the bandwidth of about 400 Hz and the steady state accuracy.

## 6. Conclusion

DE transducer combine high energy densities and multi-functional operation modes. Multilayer topologies like the DE stack-actuator considered here have also high force densities with considerable absolute deformations so that they are well-suited to be used as active skins or as end effector in soft-robotic applications. But, beside the transducer design also appropriate control and sensing algorithms are required to enable the combined actuator-sensor-operation in closed loop operation without external sensors to measure mechanical states. The design of such a self-sensing state and disturbance estimator as a universal energy control that uses the information from a novel self-sensing estimator were addressed within this contribution.

For this purpose, in section 2 the control plant comprising a DE stack-transducer fed by a bidirectional flyback converter and its model to describe the electormechanically coupled behavior was summarized. To characterize the electrical behavior the model includes the energy *U*_c,diel_ as one state variable. Based on this model subsequently a self-sensing state and disturbance estimator was developed that estimates the mechanical state of the transducer as well as an external load force by just measuring the terminal voltage and current. Due to the non-linear system behavior an EKF was used for this purpose. It allows to estimate the transducer state without any superimposed voltage excitation as used for other self-sensing approaches. The validation results have shown that almost no confinements in terms of dynamics and accuracy compared to the sensor-based estimator are obtained. The sensor-based estimator requires a measurement of the terminal voltage and the displacement.

The developed energy control uses the information provided by the self-sensing EKF for closed loop operation. Due to the behavior of the bidirectional flyback converter, that either charges or discharges a DE transducer with almost constant power when enabled, the sliding mode control approach was applied. By controlling the energy in the capacitance of the DE transducer it is possible to control the voltage, force or displacement of the transducer by using different feed-forward control structures. The setpoint energy required to achieve a certain actuator force or displacement was obtained under consideration of the static force equilibrium included in the derived model. Within the validation it was shown that a precise control of the voltage, force and displacement with high dynamics and a bandwidth of up to 400 Hz is achieved with this approach. The step response as well as the disturbance reaction yield comparable dynamics and accuracy for both the sensor-based and self-sensing control.

Although here a DE stack-transducer was considered, the developed self-sensing EKF and control approach can also be applied to other topologies well-suited for soft robotic applications like DE-based minimum energy structures or membrane actuators. The utilized bidirectional flyback converter represents an efficient and competitive converter topology and can also be used to supply any kind of DE transducer. In case of soft-bodied robots equipped with DE transducers and the mentioned converter the suggested self-sensing control approach can be used to control the impedance of the robot by applying the proposed force and displacement feed-forward controls in combination with a human-machine-interface model. If under consideration of the utilized test setup a charge of at least *q*_p_ = 5 μAs is applied, the proposed self-sensing filter can also detect collisions or interactions. This could be used e.g., in human machine interfaces or active skins, so that the control can react on these events. While for these applications the force and displacement control are most important, the voltage control could be used to avoid exceeding limitations that would cause a damage of the transducer.

## Data Availability Statement

The raw data supporting the conclusions of this manuscript will be made available upon request to JM ( juergen.maas@tu-berlin.de).

## Author Contributions

The research results included in this contribution are the outcome of TH's Ph.d. thesis that was supervised by JM.

### Conflict of Interest

The authors declare that the research was conducted in the absence of any commercial or financial relationships that could be construed as a potential conflict of interest.
